# Audiological Evaluation of Patients Taking Kanamycin for Multidrug Resistant Tuberculosis

**Published:** 2016-05

**Authors:** Vishal Sharma, Sanjeev Bhagat, Bhimsain Verma, Ravinder Singh, Surinderpal Singh

**Affiliations:** 1 *Department of Otorhinolaryngology, Government Medical College, Patiala, Punjab, India. *; 2 *Department of Pulmonary Medicine, Government Medical College, Patiala, Punjab, India. *

**Keywords:** Co-morbidity, Kanamycin, Ototoxicity, MDR-TB, Pure tone audiometry, Socioeconomic status

## Abstract

**Introduction:**

The incidence of multidrug resistant tuberculosis is increasing in developing countries. Aminoglycosides are an integral part of second-line drugs, however ototoxicity is a major limitation for their use. This study aims to determine the extent of hearing loss in patients taking one of the commonly prescribed drugs for Multidrug resistant tuberculosis (MDR-TB), Kanamycin, at a Government Medical College, Patiala, Punjab, India, which is a 1200 bed tertiary care hospital.

**Materials and Methods:**

A total of 100 patients (68 males and 32 females) with confirmed diagnosis of MDR-TB were included in this study conducted between January 2012 and February 2014. Subjects were between 15 to 60 years of age, with a mean age of 37.46 ± 10.1. Pure tone audiometry (PTA) was performed before the start of the therapy, as a baseline, and was repeated after 1 week and 6 weeks of Kanamycin use to assess hearing loss as an effect of therapy.

**Results:**

Of the 100 patients examined, ototoxicity was found in 18 subjects post therapy. Incidence of high frequency hearing loss was 2% at week 1, and 12% after 6 weeks of follow up. However, 4% of the cases developed flat loss at week 6. The hearing loss was bilateral in 13 patients and unilateral in 5 patients. Ototoxicity was more common in males (66.67%) compared to females (33.3%). Maximum cases were found in the age group of 36 to 45 years (36.8%), the majority being from a rural background (83.3%). The association with socioeconomic status (P=0.024) and co-morbid conditions like diabetes and hypertension (P=0.001) reached statistical significance.

**Conclusion:**

Lack of specific guidelines to monitor patients taking aminoglycosides makes ototoxicity a major adverse effect of their use in MDR-TB. More studies are mandated to study the risk factors associated with the development of ototoxicity and for the development of alternate drugs for the treatment of MDR-TB.

## Introduction

Tuberculosis (TB) remains a major global health problem. The latest estimates report that there were 8.6 million new TB cases in 2012 and 1.3 million TB deaths (just under 1.0 million among HIV-negative people and 0.3 million HIV-associated TB deaths) (1).WHO estimates that there were about 450,000 new (incident) multidrug resistant tuberculosis (MDR-TB) cases in the world in 2012, more than half of which occurred in China, India, and the Russian Federation (1).

MDR-TB is treated with a number of second-line drugs including the quinolones (Ciprofloxacin, Ofloxacin, and Sparfloxacin) and aminoglycosides (Streptomycin, Kanamycin, and Amikacin). These drugs are considerably more expensive and toxic than first-line drugs. The same spectrum of toxicity is seen within all members of the aminoglycoside family, the most deleterious being ototoxicity and nephrotoxicity (2).

Aminoglycoside ototoxicity is recognized by a distinctive pattern of hearing loss starting in the high frequency range (4000–8000 Hz) with lower frequencies being affected only later (3). The current data available in the literature is further confounding. On one hand, very few patients receiving aminoglycosides complain of developing hearing loss. At the same time, the reported incidence of hearing loss is up to 41% in studies in which auditory thresholds were obtained. Reasons for this discrepancy could be diverse. Patients are unlikely to complain of hearing loss until considerable damage has been done, and more importantly, there is no universal gold standard for the definition of drug-induced hearing loss (4).

Our current knowledge regarding the toxicity of Kanamycin remains extremely limited. Hence, in this study we set out to examine the incidence of ototoxicity in patients receiving Kanamycin as part of the treatment for drug resistant TB and the risk factors associated with ototoxicity, since these factors in the development of auditory toxicity are still not clearly defined.

## Materials and Methods

A total of 100 patients (68 males and 32 females) with a confirmed diagnosis of MDR-TB were included in the study, which was conducted between January 2012 and February 2014. Subjects were between 15 to 60 years of age, with a mean age of 37.46 ± 10.1. The majority of patients were from a rural background (70%). A detailed history of symptoms was taken including parameters such as difficulty in hearing, tinnitus, dizziness, vertigo, and their duration. Detailed drug history, including the doses and route of administration, was also noted. Previous exposures to any ototoxic drugs and exposure to any loud sounds in the past or occupational exposure to noise was further noted. A detailed procedure of the test was explained to all subjects and the findings were recorded on a predesigned proforma. The study was approved by the ethics committee of the institution. 

Complete otolaryngologic examination was done as part of pre-treatment clinical examination in all patients. Patients with any pre-treatment evidence of hearing loss identified while taking history, during clinical assessment (such as evidence of infective pathology in ear) or patients having conductive (A-B gap>10 dB) or sensorineural hearing loss on pure tone audiometry (PTA) were excluded. Baseline pure tone audiograms between 125 Hz and 8000 Hz were performed for all the patients. Each patient received 15 mg/kg/day of Kanamycin and audiometric evaluation was done after 1 week and 6 weeks post therapy. Kanamycin was stopped in patients complaining of or showing audiological evidence of hearing loss and was substituted with other second-line drug/drugs depending on the drug sensitivity testing.

PTA is the method of choice for testing adults and allows the testing of different frequencies and amplitudes in both ears independently. PTA was done in a sound proof room using the Elkoneda 3N3 Multi audiometer and Tecmo MT-30 headphones. The limit of normality was defined as a maximum intensity of 25 dB for all frequencies. When one or more frequencies showed values equal to or greater than 30 dB, the test was considered abnormal, even if unilaterally. The criteria used for determining ototoxicity high frequency loss (HFL) from baseline audiogram were (as per ASHA guidelines): (I) 20 dB or greater decrease at any one test frequency (4000, 6000 or 8000 KHz), (II) 10 dB or greater decrease at any two adjacent frequencies, or (III) loss of response at three consecutive frequencies (5). Flat loss was considered when in addition to HFL, the above criteria were also fulfilled in the frequencies ranging from 250 to 3000 Hz.

Incidence of hearing loss after 1 week and 6 weeks of Kanamycin therapy was calculated. Statistical analysis of the data was performed by χ2 test (chi-square test) and the Fisher exact probability test (2-tailed); p-values less than 0.05 were considered statistically significant.

## Results

Out of 100 patients examined, ototoxicity was observed in 18 subjects. Incidence of HFL in our study was 2% at week 1, and 12% after 6 weeks of follow up, while 4% of the cases developed flat loss at week 6. The hearing loss was bilateral in 13 patients and unilateral in 5 patients (Table. 1).

 Ototoxicity was more common in males (66.67%) compared to females (33.3%). The maximum cases were in the age group of 36 to 45 years (38.9%). However, there was no significant association with sex and age (P= 0.607 and 0.513 respectively). The majority of patients with hearing loss belonged to a low socioeconomic status and rural background (83.3%) while urban areas contributed only to 16.7% of cases (P= 0.024).

**Table1:** Percentage of hearing loss according to the ear involved

**Ear Involved**		**Number of Cases (n = 100)**		**Percentage**
		**High frequency loss (HFL)**	**Flat Loss**	
Bilateral		9	4	72.22%
Unilateral	Right Ear	3	0	27.78%
	Left Ear	2	0	

The association of smoking and alcohol as risk factors for the development of ototoxicity was evaluated. The percentage of hearing loss in patients with a history of smoking was 22.2% (P=0.302) while it was 19.35% in patients taking alcohol (P=0.540). The percentage of hearing loss in patients with a history of diabetes mellitus was 60% and was 36.4% with a history of hypertension (P=0.001) (Table. 2).

**Table 2:** Association of Co –Morbid Conditions with Incidence of Hearing Loss

**Co-morbid condition**	**Hearing Loss**		**Total**
	**Yes**	**No**	
Diabetes	3	2	5
Hypertension	4	7	11
None	11	73	84

## Discussion

Tuberculosis is one of the leading infectious diseases, especially in a developing country like India. More than nine million new cases are diagnosed annually. The emergence of resistance to drugs used to treat tuberculosis and particularly multi-drug resistant (MDR-TB) has become a hindrance to effective global TB control (6). 

Kanamycin, an antibiotic elaborated by Streptomyces kanamyceticus, shows activity against *Mycobacterium tuberculosis*; however, the therapy of this disease is protracted and involves the administration of large total doses of the drug. Kanamycin is more toxic to cochlea with well documented ototoxicity but it continues to be commonly used for MDR-TB in clinical settings like ours and other developing countries where cost considerations are a major factor in patient compliance (because Kanamycin is one fourth the cost of amikacin and one tenth the cost of capreomycin) (6).

The cochleotoxicity of aminoglycosides is difficult to detect and is not easily seen with any clinical symptoms. Monitoring audiological evaluations after the baseline has been recommended 1-2 times per week for patients receiving ototoxic antibiotics (7,8). However, we were not able to monitor audiological evaluations in the present study because of the costs involved and the inability of patients from distant neighborhoods to report twice a week at our center where facilities for conventional assessment of hearing are available. It is not common to find equipment for audiometry as well as trained staff at peripheral centers in a developing country like ours. Hence, the patients were only evaluated at 1 week and 6 weeks after the start of Kanamycin therapy.

It is well known that aminoglycoside ototoxicity first appears in the higher frequencies (i.e. 4000, 6000, and 8000 Hz) and later on, progresses to the lower frequencies. The majority of the patients do not complain of any hearing impairment even when the threshold shift reaches the lower frequencies (9). Our results regarding incidence of HFL (14%) were comparable to a study by Duggal et al^[6]^, which examined 26 patients who received Kanamycin and reported an incidence of HFL in 15.4% of patients while the incidence of flat loss was observed in 7.7% of patients. De Jegar et al also reported hearing loss in 20% of patients treated with Kanamycin (n=45) (10). On the contrary, Tashneem et al reported high frequency loss in 58% of cases out of which 6% developed flat loss (11). The high incidence of HFL in their study was attributed to the fact that 85% of the patients had previous exposure to Streptomycin. The short follow up of patients in our study could be the reason of less incidence of flat hearing loss as lower frequencies are usually affected after the prolonged use of aminoglycosides.

In our study, we found that the maximum number of cases of hearing loss to be in the age group of 36 to 45 years (38.9%). However, the age of the patient did not reach statistical significance (P-0.607). Moore et al and Tashneem et al had also reported a non-significant association of age with ototoxicity (P>0.05) (11,12). On the contrary, Gatell et al found a statistically significant association of age and hearing loss (P–0.02) (13). Gulbay et al reported ototoxicity to be more common in the age between 20 and 39 years (14). This difference of association with age could be attributed to the variations in the population characteristics of different studies.

India is a developing country and 70% of the population still lives in rural areas. The incidence of tuberculosis is higher in this population because of poor living conditions, overcrowding, and malnutrition. The incidence of hearing loss in patients belonging to low socioeconomic status is high in our study because patients in the rural areas may have poor compliance to drugs and hence, drug resistance is more common. This factor reached statistical significance (P–0.024) indicating that patients from a rural background are at an increased risk of developing ototoxicity. According to our knowledge, only one study has included socioeconomic status as a variable (8). However, the association with hearing loss in these patients was not evaluated. The factors contributing to this increased risk of hearing loss need to be further evaluated.

We found a significant association (P–0.001) between the presence or absence of co-morbid conditions (diabetes mellitus and hypertension) with the appearance of hearing loss. To the best of our knowledge, only one study conducted by Gatell et al has discussed the association of diabetes mellitus with ototoxicity (13), which did not find any statistical significance (P–0.44). Diabetes mellitus is emerging as a risk factor for the development of hearing impairment. The pathophysiological explanation for diabetes-related hearing loss is speculative. Diabetic complications including retinopathy, nephropathy, and peripheral arterial disease are primarily vascular in origin. Diabetic neuropathies affect peripheral sensation and various autonomic functions. The pathological changes that accompany diabetes may similarly cause injury to the vasculature or the neural system of the inner ear (15).The possibility of diabetes and aminoglycosides having a synergistic effect on cochlear hair cells cannot be ruled out. Epidemiological evidence demonstrating a relationship between diabetes and hearing impairment is just emerging and requires further investigation. 

Although several studies have systematically assessed hearing loss and analyzed risk for ototoxicity due to aminoglycosides, only a few have studied the ototoxic effects of a single aminoglyco- side and the association of risk factors in the development of hearing loss (Table. 3). 

**Table 3:** Statistical Analysis of Association of Various Risk Factors

**Factors**	**P - value**				
	**Javadi et al (2011)**	**de Jeger et al (2002)**	**Gatell et al (1987)**	**Moore et al (1984)**	**Present Study **
Age (years)	0.2	0.771	0.01	> 0.05	0.607
Sex (M/F)	0.008	0.436	0.32	> 0.05	0.513
Socioeconomic status (Rural/Urban)	-	-	-	-	0.024
Occupation	-	-	-	-	0.097
Co-morbid condition (Diabetes/hypertension)	-	-	0.44	> 0.05	0.001
Alcohol Intake	0.65	-	-	-	0.540
Smoking	0.24	-	-	-	0.302

To the best of our knowledge, this is one of the first studies that investigates the relationship between patient characteristics (socioeconomic status and co-morbid conditions) and Kanamycin induced ototoxicity in MDR-TB. Even though a positive association has been found between hearing loss and socioeconomic status and co-morbid conditions, the factors leading to it need to be further evaluated. The study is further strengthened by the fact that it includes a large sample size (n=100) wherein the role of a single aminoglycoside (Kanamycin) has been evaluated for ototoxicity. However, this study also had a few limitations, the most important being the shorter duration of patient follow up. We were unable to evaluate the relationship between ototoxicity and repeated course of treatment because our paradigm only included a fixed duration and dosage of Kanamycin. Another drawback was the lack of objectivity because we only used PTA to evaluate the hearing deficit. Otoacoustic emission (OAE) is an objective tool for the detection of hearing loss and is a much better modality for early diagnosis of ototoxicity (16). 

## Conclusion

Ototoxicity is one of the major limitations of the use of aminoglycosides in the treatment of MDR-TB. Due to the lack of specific guidelines regarding monitoring of ototoxicity, many patients report to us with advanced ototoxicity. 

This study examined the risk factors associated with ototoxicity from Kanamycin and found socioeconomic status and co-morbid conditions to be significant. However, we did not detect significance with age, gender, occupation, alcohol, or smoking. A larger and long-term study may provide better understanding in order to further identify these risk factors in greater detail. Further, with regular audiological monitoring of patients receiving Kanamycin, changes in hearing threshold can be detected at an early stage.
